# Brain Microglial Activation Increased in Glucocerebrosidase (*GBA*) Mutation Carriers without Parkinson's disease

**DOI:** 10.1002/mds.28375

**Published:** 2020-12-05

**Authors:** Stephen Mullin, Morten Gersel Stokholm, Derralyn Hughes, Atul Mehta, Peter Parbo, Rainer Hinz, Nicola Pavese, David J. Brooks, Anthony H.V. Schapira

**Affiliations:** ^1^ Department of Clinical and Movement Neurosciences, Institute of Neurology UCL London UK; ^2^ Institute of Health and Care Research University of Plymouth Peninsula School of Medicine Plymouth UK; ^3^ Department of Nuclear Medicine & PET Centre Aarhus University Hospital Aarhus Denmark; ^4^ Department of Haematology, Institute of Immunity and Transplantation UCL London UK; ^5^ Wolfson Molecular Imaging Centre University of Manchester Manchester UK; ^6^ Institute of Translational and Clinical Research Newcastle University Newcastle UK; ^7^ Lysosomal storage disease unit Royal Free Hospital London UK

**Keywords:** Parkinson's disease, microglia, substantia nigra, glucocerebrosidase, positron emission tomography

## Abstract

**Background:**

Glucocerebrosidase gene mutations are a common genetic risk factor for Parkinson's disease. They exhibit incomplete penetrance. The objective of the present study was to measure microglial activation and dopamine integrity in glucocerebrosidase gene mutation carriers without Parkinson's disease compared to controls.

**Methods:**

We performed PET scans on 9 glucocerebrosidase gene mutation carriers without Parkinson's disease and 29 age‐matched controls. We measured microglial activation as ^11^C‐(*R*)‐PK11195 binding potentials, and dopamine terminal integrity with ^18^F‐dopa influx constants.

**Results:**

The ^11^C‐(*R*)‐PK11195 binding potential was increased in the substantia nigra of glucocerebrosidase gene carriers compared with controls (Student *t* test; right, *t* = −4.45, *P* = 0.0001). Statistical parametric mapping also localized significantly increased ^11^C‐(*R*)‐PK11195 binding potential in the occipital and temporal lobes, cerebellum, hippocampus, and mesencephalon. The degree of hyposmia correlated with nigral ^11^C‐(*R*)‐PK11195 regional binding potentials (Spearman's rank, *P* = 0.0066). Mean striatal ^18^F‐dopa uptake was similar to healthy controls.

**Conclusions:**

In vivo ^11^C‐(*R*)‐PK11195 PET imaging detects neuroinflammation in brain regions susceptible to Lewy pathology in glucocerebrosidase gene mutation carriers without Parkinson's. © 2020 The Authors. *Movement Disorders* published by Wiley Periodicals LLC on behalf of International Parkinson and Movement Disorder Society

The glucocerebrosidase gene (*GBA*) encodes the lysosomal hydrolase glucocerebrosidase. In the biallelic (homozygous or compound heterozygous) state, *GBA* mutations may cause Gaucher disease (GD) which leads to glucosylceramide accumulation in visceral organs and, in a minority of cases, the central nervous system (neuronopathic GD). *GBA* mutations are the most significant genetic risk factor for Parkinson's disease (PD) and dementia with Lewy bodies (DLB)^1^, ^2^, ^3^; however, penetrance is only 10%–30%.^4^, ^5^, ^6^ PD patients carrying a *GBA* mutation have an earlier disease onset and a higher risk of dementia.^7^

At postmortem, α‐synuclein aggregations identical to those found in idiopathic PD^1^ and DLB^8^ are present in *GBA*‐PD subjects. Asymmetrically reduced striatal ^18^F‐dopa uptake,^9^, ^10^ striatal dopamine transporter binding,^11^, ^12^ and an altered striatal asymmetry index^13^ have been reported in PD patients with *GBA* mutations. Conversely ^123^I‐isoflupane dopamine transporter uptake has been demonstrated to be upregulated in non‐PD GBA carriers compared with controls and is higher in GBA PD compared to idiopathic PD cases.^14^, ^15^ *GBA* mutation carriers without PD exhibit prodromal PD features,^16^, ^17^, ^18^, ^19^ which progress with time.^20^

Glial activation has been demonstrated in postmortem PD brains.^21^, ^22^ Nigral microglial activation along with reduced striatal ^18^F‐Dopa uptake is present in idiopathic rapid eye movement sleep behavior disorder (RBD).^23^ It is also a feature of neuronopathic GD at postmortem^8^ and in GD mouse models.^24^ No studies have investigated in vivo the presence of brain microglial activation in *GBA* mutation carriers and related this to the presence of striatal dopaminergic dysfunction. We therefore measured ^11^C‐(*R*)‐PK11195 regional binding potentials (BP_ND_) and ^18^F‐dopa K_i_ in *GBA* mutation carriers without evidence of Parkinson's disease.

## Methods

### Recruitment and Clinical Assessments

Between 2015 and 2016, 9 biallelic (homozygous or compound heterozygous) or heterozygous carriers of *GBA* mutations were recruited from University College London, UK (see Table 1 for characteristics). All subjects had exons 1–11 of the *GBA* gene sequenced (Table 1). Biallelic carriers had type 1 GD, whereas heterozygous carriers were drawn from GD kindreds. No subjects met PD (UK Brain Bank) diagnostic criteria, and none were genetically related. Two of 5 GD patients were receiving enzyme replacement therapy (ERT; velaglucerase 800 IU weekly and 4000 IU monthly) and 3 of 5 substrate reduction therapy (SRT: eligustat 84 IU twice daily in 2 of 3, miglustat 300 mg once daily in 1 of 3). Both SRT and ERT were administered throughout the duration of the study. Ethical approval was obtained from London, UK (10/H0720/21), and Midtjylland, Denmark (M‐2014‐397‐14), research ethics committees.

**TABLE 1:** Characteristics of control and *GBA* carrier groups

	Biallelic *GBA* (n = 5)	Heterozygous *GBA* (n = 4)	Combined *GBA* (n = 9)	^11^C‐(R)‐PK11195 controls (n = 20)		^18^F‐Dopa controls (n = 9)	
Age, years	62.6 (2.9)	63.3 (7.7)	62.9 (2.9)	66.8 (6.0)		64.6 (3.6)	
Male, %	40.0	50.0	44.4	60.0		100.0	
UPSIT	33.6 (1.1)	31.5 (3.9)	32.7 (2.7)				
MoCA	27.4 (1.9)	27.8 (2.2)	27.6 (1.9)				
MDS UPDRS II	2.0 (2.1)	3.0 (3.6)	2.4 (2.7)				
MDS UPDRS III	12.8 (10.4)	4.5 (2.4)	9.1 (8.7)				
BDI	2.6 (2.7)	4.0 (1.4)	3.2 (2.2)				
NMSS	13.8 (9.2)	17.0 (10.4)	15.2 (9.3)				
RBDSQ	2.0 (1.9)	4.5 (2.4)	3.1 (2.4)				

Mutations of *GBA* group							
		Gaucher disease	Enzyme replacement therapy		Substrate reduction therapy		
N370s/L444P^a^		Yes	No		Yes		
N370S/IVS2 + 1^a^		No	No		Yes		
N370S/F216Y		Yes	Yes		No		
N370S/R359X^b^		Yes	No		Yes		
N370S/V447E		Yes	Yes		No		
RecNcil (L444P/A456P/V460V)^a^/wt		No	No		No		
N370S/wt		No	No		No		
N370S/wt		No	No		No		
V394L^a^/wt		No	No		No		

Clinical scores of *GBA* carriers							
Participant	MDS UPDRS II	MDS UPDRS III	MoCA	UPSIT	BDI	NMSS	RBDSQ
1	0	2	30	37	4	15	7
2	0	3	25	30	2	4	2
3	2	4	30	35	2	8	1
4	5	29	26	32	3	13	4
5	0	4	26	33	7	28	4
6	3	11	29	34	1	16	1
7	0	7	27	31	5	29	0
8	2	6	29	28	5	20	1
9	0	16	26	34	0	4	0

GBA, glucocerebrosidase; PD, Parkinson's disease; MDS UPDRS, Movement Disorder Society Unified Parkinson's Disease Rating Scale; NMSS, Non‐Motor Symptoms Scale; MMSE, Mini–Mental State Examination; MoCA, Montreal Cognitive Assessment; BDI, Beck's Depression Index; RBDSQ, REM Sleep Behavior Disorder Questionnaire.

For demographics, results are mean (SD).

^a^ Severe mutation of *GBA* carrier group.

^b^ Null mutation of *GBA* carrier group.

Each *GBA* carrier had ^11^C‐(*R*)‐PK11195 and ^18^F‐dopa PET, an MRI, and neurological examination. Prodromal PD features were rated with the University of Pennsylvania Smell Identification Test (UPSIT), Montreal cognitive assessment, RBD questionnaire (RBDSQ), PD Non‐Motor Symptoms Scale, the Movement Disorder Society Unified Parkinson's Disease Rating Scale (MDS‐UPDRS) parts II and III, and Beck's Depression Inventory.

All scans and examinations were performed at Aarhus University Hospital, Denmark. *GBA* carrier PET findings were compared with in‐house PET data from 29 age‐matched healthy controls (20 had ^11^C‐[*R*]‐PK11195 BP_ND_ PET, and 9 had ^18^F‐dopa PET) recruited for a previously published study.^25^ Assessments of control prodromal PD features were not available.

### PET and MRI

We performed prespecified region‐of‐interest (ROI) analyses comparing *GBA* mutation carriers with controls. Selected ROIs were the substantia nigra (SN), putamen, and caudate for ^11^C‐(*R*)‐PK11195 BP_ND_ and the putamen and caudate for ^18^F‐dopa K_i_. We performed statistical parametric mapping (SPM) of ^11^C‐(*R*)‐PK11195 uptake across all brain voxels. Technical details of the PET and MRI scanning and analysis procedures are available in the supplementary materials.

### Statistics

For the ROI analyses, statistical calculations and graphs were produced with Stata v14.2 software (StataCorp., College Station, TX). The ^18^F‐dopa K_i_ and ^11^C‐(*R*)‐PK11195 BP_ND_ values from specified ROIs were compared in carrier and control groups using the Student *t* test (*P* < 0.05). When there was a significant difference in ^11^C‐(*R*)‐PK11195 BP_ND_ between the *GBA* and control groups, secondary analyses correlating PD prodromal features with ^11^C‐(*R*)‐PK11195BP_ND_ were undertaken (Spearman's rank: all clinical scales were non normally distributed, *P* < 0.05). A Bonferroni correction was applied to all significant results.

## Results

### Participants

Participant characteristics are listed in Table 1. Nine *GBA* mutation carriers (5 biallelic and 4 heterozygous) were selected on the basis of their genotype and the absence of PD features. Two age‐matched control groups (20 for ^11^C‐(*R*)‐PK11195 BP_ND_ PET and 9 for ^18^F‐dopa PET) were included in the final *GBA* analysis. Some GD patients had musculoskeletal problems typical of GD reflected in raised MDS UPDRS III scores, but these were not specific for PD. This reflects the limitations of the MDS UPDRS when used in the context of non‐PD comorbidities and applied to subjects without diagnosed PD. No participants had a bradykinetic or rigid syndrome on expert examination. There were no missing data.

### Substantia Nigra ^11^C‐(R)‐PK11195 BPND Is Increased in GBA+ Individuals Compared With Controls

**FIG. 1.**
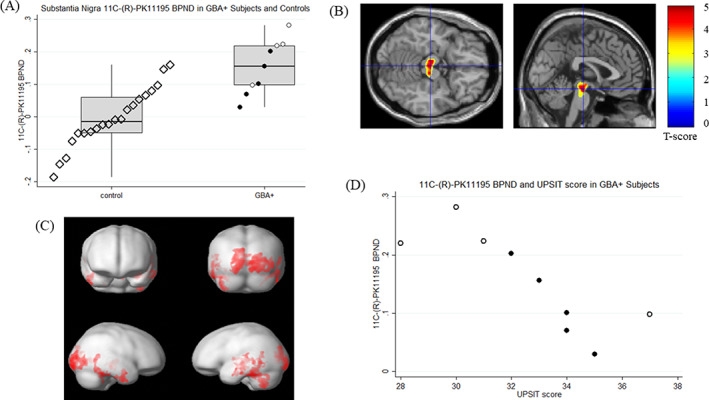
(**A**) Top left, box and dot plots of ^11^C‐PK11195 binding potential (BP_ND_) in the substantia nigra of *GBA+* heterozygous carriers (white circles), biallelic *GBA+* carriers (black circles), and controls (hollow black diamonds). Please note data points are offset across *x* axis for ease of interpretation. Middle line is median, box is interquartile range. (**B**) Top right, ^11^C‐PK11195 binding potential (BP_ND_) in *GBA* carriers > controls. Colored areas depicted on the single‐subject brain template illustrate clusters of voxels of ^11^C‐PK11195 binding potential (BP_ND_) surviving *P* < 0.05 with family‐wise error rate (FWE) correction in the brain stem region of *GBA+* carriers compared with control subjects. Non–brain stem clusters are masked. *GBA*, n = 9; controls, n = 20. (**C**) Bottom left, ^11^C PK11195 binding potential (BP_ND_) in GBA carriers > controls. Red areas depicted on the brain surface template illustrate clusters of voxels of ^11^C‐PK11195 BP_ND_ surviving *P* < 0.05 with FWE correction in cortical regions of *GBA+* carriers compared with control subjects. *GBA+*, n = 9; controls, n = 20. (**D**) Bottom right, scatterplots of ^11^C‐PK11195 BP_ND_ in the substantia nigra of *GBA+* carriers against University of Pennsylvania Smell Identification Test (UPSIT) score. *GBA+* heterozygous carriers (white), biallelic *GBA+* carriers (black).

ROI analysis localized a significant increase in mean nigral ^11^C‐(*R*)‐PK11195 BPND of the *GBA* carriers compared with controls (Student *t* test, *t* = −4.45, *P* = 0.0001; Tables S1 and S2). Statistical significance was retained after correction for multiple comparisons (Table S2). For the *GBA* mutation carriers, mean SN ^11^C‐(*R*)‐PK11195 BPND was 0.15 ± 0.08 compared with −0.01 ± 0.09 for the control group (Table S1 and Fig. 1A). Interestingly, heterozygous carriers had disproportionately higher BP_ND_ than biallelic (GD) patients (Table S1 and Fig. 1A).

### ^11^C‐(R)‐PK11195 BPND Correlates With Olfactory Deficit in GBA+ Individuals

There was a negative correlation between nigral ^11^C‐(*R*)‐PK11195 BPND and UPSIT scores in *GBA* mutation carriers (Spearman's rank, *P* = 0.0066; Table S2 and Fig. 1D), which did not survive correction for multiple comparisons (Table S2).

### Upregulated Cortical, Hippocampal, and Mesencephalon 11C‐(*R*)‐PK11195 BP_ND_ in GBA+ Group

SPM‐localized clusters of voxels with significantly increased ^11^C‐(*R*)‐PK11195 BP_ND_ in *GBA* carriers bilaterally in the occipital and temporal cortices, cerebellum, left hippocampus, and central and anterior mesencephalon (Table S3 and Fig. 1B,C). No brain regions showed reduced ^11^C‐(*R*)‐PK11195 BP_ND_ compared with controls.

### No Difference in Mean ^18^F‐Dopa K_i_ Between GBA+ and Control Participants

The *GBA* carriers showed no significant decreases in mean ^18^F‐dopa K_i_ across striatal ROIs compared with controls (Tables S1 and S2, Fig. S1). Two participants had putamen and/or caudate ^18^F‐dopa K_i_ more than 2 SDs below the control mean (Table S4). Greater variance in ^18^F‐dopa K_i_ (see Table S1) was seen in the *GBA* group (SD of 0.002 in the putamen and caudate compared with SD of 0.001 in controls). Post hoc analysis (Student *t* test) comparing the anterior, medial, and posterior putamen did not show any significant mean differences between *GBA* mutation carriers and controls.

### No Correlation Between Nigral ^11^C‐(R)‐PK11195 BP_ND_ and ^18^F‐Dopa K_i_ in GBA+ Group

There was no association between the SN ^11^C‐(*R*)‐PK11195 BP_ND_ and putamen or caudate (Table S2) ^18^F‐dopa K_i_ in the *GBA* group.

## Discussion

Our data indicate that both heterozygous and biallelic *GBA* mutation carriers can have increased ^11^C‐(*R*)‐PK11195 BP_ND_ in brain regions susceptible to Lewy body formation.^26^ It is unclear whether this is a cytotoxic or neuroprotective process. Only 10%–30% of *GBA* mutation carriers will develop PD. It is therefore unlikely that all the participants in this study will convert. Which *GBA* carriers are likely to progress to PD and the mechanisms underlying this conversion are of particular interest.

^11^C‐(*R*)‐PK11195 BP_ND_ values in the SN correlated with UPSIT scores, suggesting that those *GBA* carriers who have reduced olfactory function have higher nigral inflammation. Correlation of striatal ^11^C‐(*R*)‐PK11195 BP_ND_ with age and MDS UPDRS III score has also been shown in early PD cases.^27^

Despite mean nigral ^11^C‐(*R*)‐PK11195 BP_ND_ being increased in the *GBA* group, no significant reduction in mean putamen ^18^F‐dopa uptake was seen. It is known that ^18^F‐dopa lacks the sensitivity to detect early dopaminergic dysfunction because of compensatory upregulation of dopa decarboxylase in the remaining terminals. Early reductions may be better detected with dopamine transporter markers.^28^, ^29^ Our finding of normal striatal F‐dopa uptake in *GBA* carriers may not necessarily equate to normal dopamine terminal function, although no *GBA* carrier exhibited clinical features of PD.

Interestingly ^18^F‐dopa Ki was more variable in the *GBA* group compared with controls. Recently, 184 nonmanifesting *GBA* carriers were reported to have increased dopamine transporter binding across striatal regions.^15^ This is in line with an increase in striatal ^18^F‐dopa K_i_ found in a portion of our *GBA*+ cases. It has been reported that ^11^C‐(*R*)‐PK11195 binding to microglia “burns out” as amyloidosis in early Alzheimer's disease advances^30^ but increases again as tau tangles form.^31^, ^32^ A biphasic trajectory could explain the lack of correlation between ^18^F‐dopa K_i_ and ^11^C‐(*R*)‐PK11195 BP_ND_ in our data set.

### Limitations

The relatively small sample size, its cross‐sectional design, and the unknown future disease status of *GBA* mutation carriers are limitations. We acknowledge that *GBA* mutations exhibit a variable penetrance and phenotype, in terms of both PD and GD. Reproducing these results in larger (ideally prospective) and more genotypically and phenotypically homogenous cohorts is needed. Nevertheless, we believe these are important and highly relevant pilot data that will inform the design of future studies.

The ^11^C‐PK11195 BP_ND_ has high nonspecific binding, which provides a lower specific‐to‐background PET signal ratio than newer markers of activated microglia; therefore, our results may underestimate glial activation. This study used ^11^C‐(*R*)‐PK11195 BP_ND_ as a marker of the translocator protein (TSPO) expressed by the mitochondria of activated microglia, and, in contrast to newer TSPO tracers available, the binding is not influenced by the polymorphism of the TSPO expressed by individuals. The limitations of supervised cluster analysis in conditions with possible widespread microglial activation should also be acknowledged, as it could lead to an underestimation of ^11^C‐(*R*)‐PK11195 BP_ND_, particularly in small ROIs.

Three of 5 and 2 of 5 subjects were taking substrate reduction therapy or enzyme replacement therapy (ERT), respectively. The former is under evaluation as a PD neuroprotective agent (clinicaltrials.gov, NCT02906020). ERT is not thought to cross the blood–brain barrier, although 1 report suggests a portion may.^33^ We cannot exclude the possibility that the reduced nigral and putamen ^11^C‐(*R*)‐PK11195 BP_ND_ in biallelic compared with heterozygous cases could represent suppression of glial activation by these drugs.

## Conclusions

Our findings indicate that *GBA* mutations are associated with microglial activation in Lewy‐susceptible brain regions in subjects without either a prodromal or clinical diagnosis of PD. Further studies are required to assess whether ^11^C‐(*R*)‐PK11195 BP_ND_ PET, (with or without additional biomarkers) can predict GBA carrier conversion to PD and striatal dopamine loss.

## Author Contributions

The study was designed by S.M., M.S., A.S., D.B., and N.P. Patient identification and recruitment were carried out by S.M., A.S., A.M., and D.H. Imaging was carried out by M.S., R.H., and P.P.. Image analysis was carried out by M.S. Data analysis was carried out by S.M. The article was primarily written by S.M., M.S., and A.S. with contributions from A.M., D.H., N.P., and D.B. and reviewed by all the authors.

## Full Financial Disclosures for the Past 12 Months

S.M. has received grant funding from the National Institute of Health Research, the Engineering and Physical Sciences Research Council, and fees for consulting from GLG consulting, Horama and Centogene. D.H. has received fees for research from Sanofi and Takeda, administered through UCL consultants with benefits to research in lysosomal storage diseases. D.B. has received grant funding from Horizon 2020, Danish Council for Independent Research, and Lundbeck Foundation. In the last 12 months A.H.V.S. has received funding from the Medical Research Council, H2020 Parkinson's UK, and the Cure Parkinson Trust. He is an employee of UCL. He has served as a consultant for Prevail Therapeutics. N.P. has received grants funding from the Independent Research Fund Denmark, Danish Parkinson's Disease Association, Parkinson's UK, MRC – Center of Excellence in Neurodegeneration (CoEN) network award, GE Healthcare Grant, Multiple System Atrophy Trust, Weston EU Joint Program Neurodegenerative Disease Research (JPND), EU Horizon 2020 research and innovation program, Italian Ministry of Health, and Honoria for consultancy from Britannia, Boston Scientific, Benevolent AI, and Bial. D.J.B. has received grant funding from the Independent Research Fund Denmark, Lundbeck Foundation, Danish Parkinson's Disease and Alzheimer's Associations, Horizon 2020, and GE Healthcare. M.S., P.P., and A.M. have nothing to disclose.

## Data and Materials Availability

Study data are available on reasonable request.

## Supporting information


**Table S1.** Summary table of positron emission tomography (PET) binding potentials (BP_ND_) for ^11^C‐(*R*)‐PK11195 and influx constants (K_i_) for ^18^F‐dopa in control and *GBA+* groups
**Table S2.** Summary table of analyses
**Table S3.** Summary table of statistically significant statistical parametric mapping findings for ^11^C‐(*R*)‐PK11195 regional binding potentials (BP_ND_)
**Table S4.** The ^11^C‐(*R*)‐PK11195 regional binding potentials (BP_ND_) and ^18^F‐dopa influx constants (K_i_) greater than 2 SD below the control group mean with UPSIT and MDS UPDRS III assessment scores in *GBA+* group
**Figure S1**. Box and dot plots of ^18^F‐dopa K_i_ in (A) the caudates of heterozygous GBA+ carriers (white circles), biallelic GBA+ carriers (black circles), and controls (hollow black diamonds). Please note data points are offset across *x* axis for ease of interpretation; and (B) in the putamina of heterozygous GBA+ carriers (white circles), biallelic GBA+ carriers (black circles), and controls (hollow black diamond). Please note data points are offset across *x* axis for ease of interpretation. For (A) and (B), middle line is median, box is interquartile range.
