# Supplementary material for: Brain Microglial Activation Increased in Glucocerebrosidase (GBA) Mutation Carriers without Parkinson's disease
Source: Mov Disord. 2020 Dec 5;36(3):774–9. doi: 10.1002/mds.28375 (PMC8048428; doi:10.1002/mds.28375)
Supplement: Supplementary file 1 — Table S1. Summary table of positron emission tomography (PET) binding potentials (BPND) for 11C‐(R)‐PK11195 and influx constants (Ki) for 18F‐dopa in control and GBA+ groups Table S2. Summary table of analyses Table S3. Summary table of statistically significant statistical parametric mapping findings for 11C‐(R)‐PK11195 regional binding potentials (BPND) Table S4. The 11C‐(R)‐PK11195 regional binding potentials (BPND) and 18F‐dopa influx constants (Ki) greater than 2 SD below the control group mean with UPSIT and MDS UPDRS III assessment scores in GBA+ group Figure S1. Box and dot plots of 18F‐dopa Ki in (A) the caudates of heterozygous GBA+ carriers (white circles), biallelic GBA+ carriers (black circles), and controls (hollow black diamonds). Please note data points are offset across x axis for ease of interpretation; and (B) in the putamina of heterozygous GBA+ carriers (white circles), biallelic GBA+ carriers (black circles), and controls (hollow black diamond). Please note data points are offset across x axis for ease of interpretation. For (A) and (B), middle line is median, box is interquartile range. [file MDS-36-774-s001.docx]

**Supplementary materials**

**Methods**

*PET and MRI*

PET and MRI were performed as previously described (Stokholm et al. 2017). PET was performed using an ECAT HRRT CTI/Siemens camera following an intravenous injection of tracer and dynamic 3D PET images were obtained in list mode for 60.5 (^11^C-(R)-PK11195) and 94.5 (^18^F-DOPA) minutes and subsequently rebinned into 24 and 26 dynamic time frames. One hour prior to ^18^F-DOPA PET an oral 150 mg dose of Carbidopa was administered. Both image series were reconstructed using 3D OSEM (ordered subsets expectation maximization) and resolution recovery modelling (PSF) with 10 iterations and 16 subsets. Reconstructed images comprised 207 transaxial image slices with a 1.22 mm voxel size. Each study participant had T1-weighted 3D MRI to co-register PET images (3T MAGNETOM Skyra or Trio, Siemens Healthcare). No participants were receiving dopaminergic therapy or medications that could interfere with ^11^C-*(R)*-PK11195 binding.

## *Image analysis*

First, whole brain parametric maps of regional binding potentials and influx constants at a voxel level were generated for the ^11^C-*(R)*-PK11195BP_ND_ and ^18^F-DOPA PET images, respectively. Parametric BP_ND_ maps of ^11^C-*(R)*-PK11195 PET were computed using the simplified reference tissue model and a supervised cluster analysis to define a reference cluster containing only non-specific signal for each individual and extract a time activity curve for the reference tissue input function (Turkheimer et al. 2007). Similarity between ^11^C-*(R)*-PK11195 time activity curves were examined with a repeated measurement analysis (*X*^2^ p>0.05). The ^18^F-DOPA parametric maps of influx Ki were generated at a voxel level with the Patlak graphical approach using occipital lobe grey matter as a reference tissue input function. The equilibration time t’ was set to 25 minutes. All parametric maps were transformed into standard Montreal Neurological Institute (MNI) stereotaxic space using Statistical Parametric Mapping (SPM12; Wellcome Trust Centre for Neuroimaging, London, UK).

*Optimisation to reduce background ^11^C-(R)-PK11195BP_ND_ signal interference*

Motion control was assured by monitoring the patient's head during the scan (making periodic checks that the laser beam is located on the pen marks of the patients face). A between frame motion correction was not be applied to the dynamic time frames as cortical signal is too noisy to allow for a reliable image based head motion correction.

In the absence of overt brain atrophy in the clinical cohort we did not need to apply partial volume correction in this study.

The HRRT scanner obtains brain PET images with a high resolution which reduces partial volume effect. This makes it more accurate in the sampling of smaller brain structures compared to a regular PET-CT system (Heiss et al. 2004).

Additionally, each subject had a high resolution T1-weighted MP2RAGE sequence MRI performed that was used to delineate regions of interest, which further reduces the partial volume effect. Partial volume effect is due to the limited spatial resolution of the imaging system, which gives a three-dimensional blurring of the image resulting in a spillover between regions. Therefore, the image of a small structure / source is a larger but dimmer source, which results in a smaller amount of radioactivity per volume unit (underestimating true binding/uptake).

The image was sampled on a voxel grid. The contours of the voxels do not match the contours of the structure of interest / tracer distribution, therefore, many voxels will contain different types of tissue. This means that the signal in each voxel is a mean value of the signal intensities from each underlying structure in this voxel (Soret, Bacharach, and Buvat 2007).

*Regions of interest (ROI) analysis*

The PNEURO module of PMOD v 3.6 software (PMOD technologies Ltd. Switzerland) was used to define and sample regions of interest as previously described (Stokholm et al. 2017). The putamen and later the caudate are sites where dopaminergic loss can be visualised with ^18^F-DOPA PET, so these were selected as the ROIs. ^11^C-*(R)*-PK11195 binds to the translocator protein expressed by activated microglial cells (Banati 2002). Increased ^11^C-*(R)*-PK11195 BP_ND_ has been previously reported in the basal ganglia of early PD cases so the putamen, caudate and substantia nigra (SN) were selected as ROIs. All ROIs were predefined prior to Statistical parametric mapping (SPM).

*Statistical Parametric Mapping (SPM) analysis*

SPM (SPM12; Wellcome Trust Centre for Neuroimaging, London, UK) was used to localise voxel level significant changes in mean ^11^C-*(R)*-PK11195BP_ND_ in *GBA* carriers compared with control subjects. The parametric ^11^C-*(R)*-PK11195BP_ND_ images of the *GBA* carriers and controls were smoothed to 6 mm full width at half maximum with a Gaussian kernel to reduce inter-subject variability and interrogated with a two-tailed Student’s t-test. We considered significant findings as voxels with an initial threshold of p<0.01 with a cluster extent of 50 voxels with post hoc FWE-correction (p<0.05) at the cluster level.

**References**

Banati, Richard B. 2002. “Visualising Microglial Activation in Vivo..” *Glia* 40 (2). Wiley Subscription Services, Inc., A Wiley Company: 206–17. doi:10.1002/glia.10144.

Heiss, Wolf-Dieter, Birgit Habedank, Johannes Christian Klein, Karl Herholz, Klaus Wienhard, Mark Lenox, and Ron Nutt. 2004. “Metabolic Rates in Small Brain Nuclei Determined by High-Resolution PET..” *Journal of Nuclear Medicine : Official Publication, Society of Nuclear Medicine* 45 (11): 1811–15.

Soret, Marine, Stephen L Bacharach, and Irène Buvat. 2007. “Partial-Volume Effect in PET Tumor Imaging..” *Journal of Nuclear Medicine : Official Publication, Society of Nuclear Medicine* 48 (6): 932–45. doi:10.2967/jnumed.106.035774.

Stokholm, Morten Gersel, Alex Iranzo, Karen Østergaard, Mónica Serradell, Marit Otto, Kristina Bacher Svendsen, Alicia Garrido, et al. 2017. “Assessment of Neuroinflammation in Patients with Idiopathic Rapid-Eye-Movement Sleep Behaviour Disorder: a Case-Control Study..” *Lancet Neurology* 16 (10): 789–96. doi:10.1016/S1474-4422(17)30173-4.

Turkheimer, Federico, Paul Edison, Nicola Pavese, federico roncaroli, Alexander Anderson, Alexander Hammers, Alexander Gerhard, Rainer Hinz, Y K Tai, and David Brooks. 2007. “Reference and Target Region Modeling of [11C]-(R)-PK11195 Brain Studies.” *Journal of Nuclear Medicine : Official Publication, Society of Nuclear Medicine*, January, 1–11.

| **Supplementary table 1. Summary table of Positron Emission tomography (PET) binding potentials (BP_ND_) for ^11^C-*(R)*-PK11195 and influx constants (Ki) for ^18^F-DOPA in control and *GBA+* groups** | | | | |
| --- | --- | --- | --- | --- |
| **^11^C-*(R)*-PK11195 regional binding potentials (BP_ND_)** | | | | |
|  | **CONTROL** | ***GBA+* COMBINED** | **HETEROZYGOUS *GBA+*** | **BI-ALLELIC *GBA+* (GAUCHER DISEASE)** |
| **n** | **n=20** | **n=9** | **n=4** | **n=5** |
| **Substantia nigra** | -0.006 (0.092) | 0.154 (0.083) | 0.206 (0.077) | 0.112 (0.068) |
| **Putamen** | 0.080 (0.112) | 0.142 (0.112) | 0.165 (0.091) | 0.142 (0.112) |
| **Caudate** | -0.163 (0.137) | -0.137 (0.079) | -0.150 (0.076) | -0.127 (0.088) |
| **^18^F-DOPA influx constant (Ki)** | | | | |
|  | **CONTROL** | ***GBA+* COMBINED** | **HETEROZYGOUS *GBA+*** | **BI-ALLELIC *GBA+* (GAUCHER DISEASE)** |
| **n** | **n=9** | **n=9** | **n=4** | **n=5** |
| **Putamen** | 0.130 (0.001) | 0.013 (0.002) | 0.122 (0.001) | 0.136 (0.002) |
| **Caudate** | 0.113 (0.001) | 0.112 (0.002) | 0.011 (0.001) | 0.121 (0.003) |
| Results are mean (SD). *GBA* Glucocerebrosidase gene. | | | | |

| **Supplementary table 2. Summary table of analyses** | | | | |
| --- | --- | --- | --- | --- |
| **Corrected and uncorrected p values for comparison (student’s t test* or Spearman’s rank**) of ^18^F-DOPA Ki and ^11^C-*(R)*-PK11195 BP_ND_ in *GBA* carrier and control group (p<0.05). Bonferroni correction factor of 2.** | | | | |
|  | **^18^F-DOPA** | **^11^C-*(R)*-PK11195** | | |
|  | *uncorrected* | *uncorrected* | | *corrected* |
| **N** | **n=18 (9 *GBA+*, 9 control)** | **n=29 (9 *GBA+*, 20 control)** | | **n=29 (9 *GBA+*, 20 control)** |
| **Substantia nigra*** | - | **p=0.0001** | | **P=0.0009** |
| **Putamen*** | p=0.9081 | p=0.1805 | | - |
| **Caudate*** | p=0.6640 | p=0.6095 | | - |
| **Corrected and uncorrected p values for correlation of prodromal PD feature score with substantia nigra ^11^C-*(R)*-PK11195 BP_ND_ in combined *GBA* carrier group. Bonferroni correction factor of 9. (n=29, 9 *GBA+*, 20 control)** | | | | |
|  | uncorrected | | corrected | |
| **UPSIT **** | **p=0.0066** | | p=0.0594 | |
| **MoCa **** | p=0.1132 | | - | |
| **RBDSQ**** | p=0.4082 | | - | |
| **BDI **** | p=0.3045 | | - | |
| **MDS UPDRS II**** | p=0.3413 | | - | |
| **MDS UPDRS III **** | p=0.9353 | | - | |
| **NMSS**** | p=0.5135 | | - | |
| *GBA*: glucocerebrosidase, PD: Parkinson disease, MDS UPDRS: Movement disorders society unified Parkinson disease rating scale, NMSS: Non motor symptoms scale, MoCa: Montreal cognitive assessment, BDI: Beck’s depression index, RBDSQ REM sleep behaviour disorder questionnaire. UPSIT - University of Pennsylvania smell identification test | | | | |

| **Supplementary table 3. Summary table of statistically significant statistical parametric mapping findings for ^11^C-*(R)*-PK11195 regional binding potentials (BP_ND_)** | | | | | | | |
| --- | --- | --- | --- | --- | --- | --- | --- |
| **Region** | **Peak-level** | | | | **Cluster-level** | | |
| ***GBA* carriers (n=9) compared to controls (n=20)** | | | | | | | |
|  | **T-score** | **Coordinates** | | | **p-value** | **p-value** | **Cluster size** |
|  |  | **X** | **Z** | **Y** | ***uncorrected*** | ***FWE-corrected at cluster level*** | ***(voxels)*** |
| Right visual associative cortex | 6.18 | 18 | -98 | -9 | **p < 0.001** | **p < 0.001** | 23922 |
| Right inferior temporal cortex | 5.84 | 47 | -43 | -26 | **p < 0.001** | **p = 0.008** | 7119 |
| Left inferior temporal cortex | 5.35 | -59 | -38 | -25 | **p < 0.001** | **p < 0.001** | 18845 |
| Left substantia nigra | 4.92 | -4 | -17 | -19 | **p < 0.001** | **p < 0.001** | 10753 |
| BP_ND_ - regional binding potentials, *GBA* Glucocerebrosidase gene. | | | | | | | |

| **Supplementary table 4. ^11^C-*(R)*-PK11195 regional binding potentials (BP_ND_) and ^18^F-DOPA influx constants (Ki) greater than than 2 SD below the control group mean with UPSIT and MDS UPDRS III assessment scores in *GBA+* group** | | | | |
| --- | --- | --- | --- | --- |
| **^Participant^** | **^18^F-DOPA Ki caudate** | **^18^F-DOPA Ki putamen** | **UPSIT** | **MDS UPDRS III** |
| 1 | - | -- | 37 | 2 |
| 2 | **-** | **-** | 30 | 3 |
| 3 | **-** | **-** | 35 | 4 |
| 4 | - | **-** | 32 | 29 |
| 5 | - | **-** | 33 | 4 |
| 6 | **>2 SD** | **>2 SD** | 34 | 11 |
| 7 | **-** | **-** | 31 | 7 |
| 8 | **-** | **-** | 28 | 6 |
| 9 | **-** | **>2 SD** | 34 | 16 |
| SD – standard deviation, MDS UPDRS: Movement disorders society unified Parkinson disease rating scale, UPSIT - University of Pennsylvania smell identification test, BP_ND_ - regional binding potentials, Ki - influx constants, *GBA* Glucocerebrosidase gene. | | | | |
